# Argentine ant extract induces an *osm-9* dependent chemotaxis response in *C. elegans*

**DOI:** 10.17912/micropub.biology.000745

**Published:** 2023-03-14

**Authors:** Sebastian A. Alfonso, Daniel Arango Sumano, Dhruv A. Bhatt, Aidan B. Cullen, Cyrus M. Hajian, Winnie Huang, Emma L. Jaeger, Emily Li, A. Kaile Maske, Emma G. Offenberg, Vy Ta, Waymon W. Whiting, Grace T. Adebogun, Annabelle E. Bachmann, Ashlyn A. Callan, Ummara Khan, Amaris R. Lewis, Alexa C. Pollock, Dave Ramirez, Nicole Bradon, Katherine Fiocca, Lauren E. Cote, Maria D. Sallee, Jordan McKinney, Lauren A. O'Connell

**Affiliations:** 1 BIO161 Organismal Biology Lab, Stanford University, Stanford, California, United States; 2 Department of Biology, Stanford University, Stanford, California, United States

## Abstract

Many ant species are equipped with chemical defenses, although how these compounds impact nervous system function is unclear. Here, we examined the utility of *Caenorhabditis elegans* chemotaxis assays for investigating how ant chemical defense compounds are detected by heterospecific nervous systems. We found that *C. elegans* respond to extracts from the invasive Argentine Ant ( *Linepithema humile* ) and the *osm-9* ion channel is required for this response. Divergent strains varied in their response to *L. humile* extracts, suggesting genetic variation underlying chemotactic responses. These experiments were conducted by an undergraduate laboratory course, highlighting how *C. elegans* chemotaxis assays in a classroom setting can provide genuine research experiences and reveal new insights into interspecies interactions.

**Figure 1. Argentine Ant ( Linepithema humile ) extracts induce an osm-9 dependent chemotaxis response in C. elegans .**
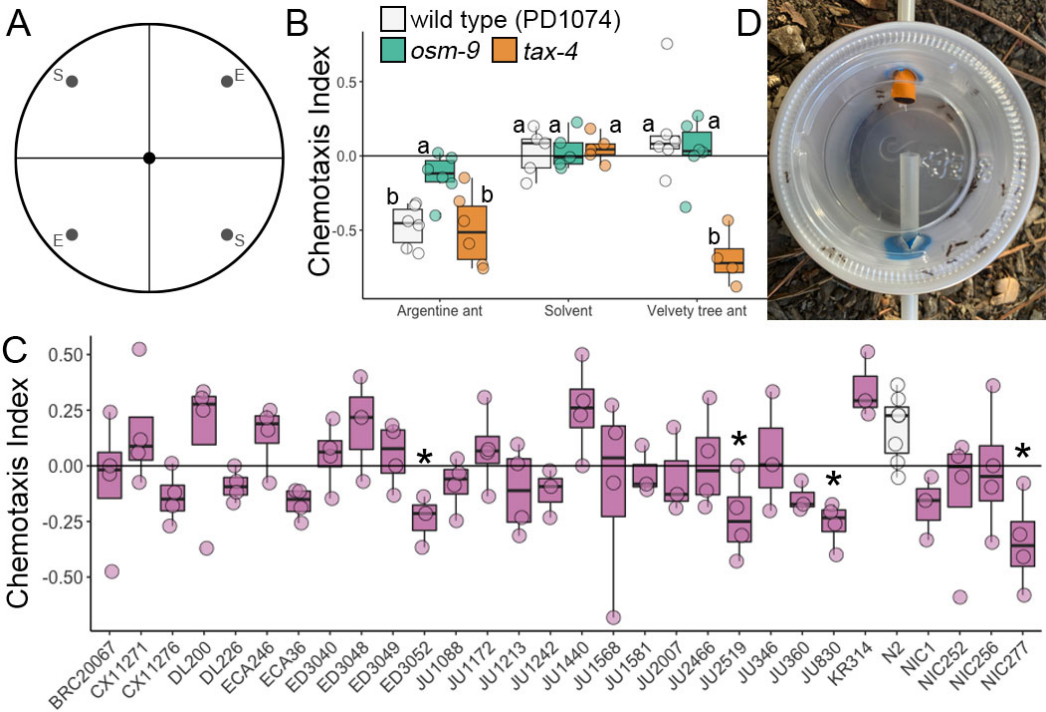
**(A)** Chemotaxis assays were performed on circular plates divided into quadrants, where worms placed in the center were exposed to ant extracts (E) and solvent (S). **(B)** The chemotaxis response of wild-type worms ( PD1074 , white), *osm-9 ( ky10 )* worms (orange), and *tax-4 ( p678 )* worms (blue) were tested in response to Argentine ant ( *Linepithema humile)* and Velvety tree ant ( *Liometopum occidentale* ) extracts. Wild-type PD1074 and *tax-4* knockout worms, but not *osm-9* knockout worms, were repelled by Argentine ant extracts. Wild-type PD1074 and *osm-9* knockout worms did not respond to Velvety tree ant extracts, whereas *tax-4* knockout worms were repelled. Groups not connected by the same letter are significantly different. **(C)** The response to Argentine ant extract was measured across a divergent strain set. Stars indicate significant differences between genetically diverse strains (purple) from wild type ( N2 , white). (D) Students constructed simple aspirators to collect ants.

## Description

Ants use a diverse array of chemicals for communication, such as identifying nestmates or as chemical defense against competitors (Fox and Adams, 2022; Schmidt, 1986). However, the receptors that bind these compounds and the classes of neurons that respond to these chemical messengers to influence behavior are not well understood, as probing the neurogenetic basis of ant chemical communication remains difficult (but see (Trible et al., 2017)). Here, we explore (1) using *C. elegans* chemotaxis assays as a simple screen for identifying genes and neurons responsive to ant chemical defenses and (2) using this approach in an undergraduate classroom setting to engage students in authentic scientific research. Chemotaxis assays using *C. elegans* are a convenient and practical approach, as this species has a short generation time, are easy to culture in large quantities, have many readily available genetic tools, and rely on chemosensation to navigate their environment (Bargmann, 2006; Corsi et al., 2015). Specifically, *C. elegans* chemosensory neurons can be silenced by removing essential ion channels such as OSM-9 (Colbert et al., 1997) or TAX-4 (Komatsu et al., 1996), allowing for relatively simple chemotaxis mutant screens that quickly identify a subset of neurons important for chemosensation of various compounds. These features render *C. elegans* a potentially promising organism for screening ant-derived compounds for behavior-altering molecules, determining the neural and molecular mechanisms underlying the behavioral response, and conducting these experiments in an undergraduate laboratory classroom.

To examine the influence of ant-derived compounds on *C. elegans* chemotaxis behavior, we tested the response of *osm-9 ( ky10 )* (Colbert et al., 1997), *tax-4 ( p678 ) (* Komatsu et al., 1996), and wild-type worms ( PD1074 , (Yoshimura et al., 2019)) to extracts derived from two different ant species: the invasive Argentine ant ( *Linepithema humile)* and the native Velvety tree ant ( *Liometopum occidentale* ) (Figure 1A-B). We found a significant interaction between strains and compounds (2-way ANOVA, Strain*Compound: F(4) = 9.3459, p < 0.001). The strains did not differ in their response to solvent, but there were significant differences in responses to the ant extracts. PD1074 animals were indifferent to solvent and extracts from the Velvety tree ants but avoided Argentine ant extracts ( PD1074 : solvent vs Argentine ant extract, t(40) = −4.223, p = 0.003). Argentine ant extracts also repelled *tax-4* mutants ( *tax-4 :* solvent vs Argentine ant extract, t(40) = −4.615, p = 0.002), but not the *osm-9* mutants ( *osm-9 :* solvent vs Argentine ant extract, t(40) = −1.434, p = 0.239). Within groups exposed to Argentine ant compounds, the response of *osm-9* mutants was significantly different from both PD1074 (t(40) = −2.990, p = 0.010) and *tax-4* mutants (t(40) = 3.189, p = 0.006). Together, these data suggest that *C. elegans* sense Argentine ant extracts and *osm-9* -expressing chemosensory neurons likely mediate this response. As Argentine ants are an invasive species, their chemicals have been extensively studied and include fatty acids, hydrocarbons, and defensive chemicals such as the terpenoid iridomyrmecin (Cavill and Houghton, 1974; Cavill et al., 1980, 1976), that are used in intra- and inter-species interactions (Welzel et al., 2018). Future experiments could include the presentation of extract fractions or commercially available extract compounds to examine which components influence *C. elegans* behavior.

Extracts of the Velvety tree ant showed a different pattern of behavioral response, in which *tax-4* mutants were repelled by the extracts ( *tax-4 :* solvent vs Velvety tree ant extract, t(40) = −5.645, p < 0.001), but *osm-9* mutants ( *osm-9 :* solvent vs Velvety tree ant extract, t(40) = 0.018, p = 0.985) and the wild type ( PD1074 : solvent vs Velvety tree ant extract, t(40) = −1.096, p = 0.373) were not. Within groups exposed to Velvety tree ant compounds, the response of *tax-4* mutants was significantly different from both PD1074 (t(40) = 6.705, p < 0.001) and *osm-9* mutants (t(40) = 5.718, p < 0.001). As *tax-4* and *osm-9* are co-expressed in only a subset of chemosensory neurons, it is possible that co-expression masks neural responses to exogenous compounds and that a behavioral phenotype is only observed when *tax-4* is removed. Inclusion of a double knockout of both *tax-4* and *osm-9* in future experiments would help clarify if there are compensatory relationships between *osm-9* and *tax-4* chemosensory neurons that would lead to avoidance in *tax-4* mutants, but not in the PD1074 strain. In addition, Velvety tree ants have a variety of hydrocarbons and fatty acids present in their extracts but lack the terpenoid iridomyrmecin present in Argentine ants (Moskowitz et al., 2022). Fractionation of Velvety tree ant extracts for future chemotaxis experiments would be the next step towards identifying compounds that elicit avoidance.

We next tested whether there is genetic variation in chemotaxis responses elicited by ant extracts by comparing genetically diverse *C. elegans* strains from the CeNDR collection (Cook et al., 2017) (Figure 1C). We focused on extracts of Argentine ants since it elicited a strong response in wild-type worms in the previous assay, and because Argentine ants are highly abundant and easily collected by undergraduate students (see Methods). Across thirty strains, including an N2 strain, there was a significant effect of Argentine ant extract on chemotaxis behavior (ANOVA, F(29) = 2.3364, p = 0.001). Four strains showed a significantly different response when compared to N2 , including ED3052 (t(84) = −2.862, p = 0.039), JU2519 (t(84) = −3.091, p = 0.026), JU830 (t(84) = −3.309, p = 0.020), and NIC277 (t(84) = −3.956, p = 0.004). Notably, the N2 strain responded differently than the PD1074 strain from the previous experiment. It is possible that genetic differences between PD1074 and other N2 strains underlie the different responses to Argentine ant extracts, as N2 strains propagated by various labs can be genetically variable whereas PD1074 is a derived clonal strain that is genetically homogenous (Yoshimura et al., 2019). Alternatively, this difference may be due to the Argentine ant extract being different across experiments, as the extracts were prepared at different times. The concentration of compounds likely varied across different preparations, which could have induced opposing chemotaxis results (Yoshida et al., 2012). Moreover, differences in extract composition could influence worm chemotaxis responses, as ant diet, which can be different across colonies or seasons, can influence ant chemistry (Liang and Silverman, 2000). Regardless, the diversity in chemotaxis responses across strains suggests that future genome-wide association studies could identify additional molecular factors important in this behavioral response (Cook et al., 2017).

In summary, we have shown that extracts of the Argentine ant influence *C. elegans* chemotaxis, *osm-9* chemosensory neurons likely mediate this response, and there are variable responses to Argentine ant extracts across divergent *C. elegans* strains. Moreover, we have uncovered a complex interaction in chemotaxis responses to extracts from Velvety tree ants. Future studies will include more mutant and divergent worm strains and more ant species with consistent collection and extraction methods that reduce response variability. Importantly, this work establishes the feasibility of using *C. elegans* chemotaxis assays as a tool for learning more about the biochemistry mediating interspecies interactions within classroom undergraduate research experience courses.

## Methods

*Worm Strains*

Strains were obtained from the Caenorhabditis Genetics Center (CGC) at the University of Minnesota and the *Caenorhabditis elegans* Natural Diversity Resource CeNDR at Northwestern University (see Reagents). Animals were maintained in 20°C incubators and nematodes were synchronized by bleaching adults to obtain eggs. Roughly 300-500 eggs were pipetted onto 55 mm Nematode Growth Media plates spread with OP50 *E. coli* . NGM plates were made as described (Stiernagle, 2006) with the addition of uracil at 2μg/mL. Hatched eggs were kept at 20°C for roughly 3 days when the population reached a young adult stage and were used for chemotaxis assays.

*Ant extracts*

The invasive Argentine ant ( *Linepithema humile)* and the Velvety tree ant ( *Liometopum occidentale* ) were collected on the Stanford University campus. In the first screening set, ant extracts that had been previously collected and analyzed with gas chromatography / mass spectrometry were used, as detailed in Moskowitz et al. (2022). To acquire more Argentine ant extract for the divergent screen, students built aspirators to collect ants using plastic ketchup cups, plastic straws, putty, and strips of pantyhose fabric (Figure 1D). After collection, ants were incubated at -80C for 15 minutes and then placed in methanol for 24 hours at 20C. Ants were removed from methanol samples before evaporation under a constant flow of nitrogen gas to dryness. Evaporated ant samples were then resuspended in dimethyl sulfoxide (DMSO).

*Chemotaxis Assays*

Undergraduate students in a laboratory course performed chemotaxis assays while unaware of compound or strain being tested until data was submitted to the instructor. Chemotaxis plates [5mM KPO _4_ (pH 6), 1mM CaCl _2_ , 1mM MgSO _4_ , 2% agar] were divided into four quadrants (Figure 1A). Compounds (5 µL, ant extract concentration unknown) were placed on dots located in two non-adjacent quadrants (E, experimental) while 5 µL of DMSO was placed on dots in the other two non-adjacent quadrants (S, solvent). Plates were then incubated for 30 minutes to allow for the establishment of a chemical gradient. During this incubation period, worms were removed from their plate and washed three times with Chemotaxis Assay Buffer [5mM KPO _4_ (pH 6), 1mM CaCl _2_ , 1mM MgSO _4_ ]. Following compound incubation, 2 µL 0.5 M sodium azide solution was applied to each of the quadrant dots to serve as a worm paralytic. Then, roughly 100 worms were placed in the center of each plate and the excess buffer was removed using a KimWipe. Worms were allowed to roam the plate for one hour and then were counted manually in each quadrant under a dissecting microscope using a tally counter. Worms in the center dot of overlapping quadrants were not counted for any quadrant but were included in the total number of worms. Eighteen students conducted all the experiments and each assay was replicated 3-6 times.

*Data Analysis*

The Chemotaxis Index (CI) was calculated for each plate: CI = (Number of worms in the two experimental quadrants – Number of worms in the two solvent quadrants) / Total number of worms on the entire plate. Thus, a positive CI indicates attraction and a negative CI indicates repulsion to the experimental compounds. Plates were removed from the data set prior to analysis if there were less than 20 worms on the plate or if a student noted a technical error in the plate setup, such as mistakes in pipetting compounds or worms onto the appropriate locations.

Data analysis and visualization were performed in R (version 4.1.2). For the screen with two ant species and three worm strains, a 2-way ANOVA was used to detect significant differences between groups, with chemotaxis index as dependent variable and compound, worm strain, and their interaction as independent variables. For the divergent screen, a one-way ANOVA was used to detect significant differences across groups, with chemotaxis index as dependent variable and worm strain as the independent variables. Parametric assumptions were met by both datasets, including homogeneity of variance confirmed with Levene’s test (leveneTest) and normality of residuals (visualized with qqp functions). Posthoc analyses were performed using emmeans (version 1.7.2) and grafify (version 3.0.0) packages with false discovery rate (fdr) adjustment of p-values to account for multiple testing. Pairwise posthoc tests were run for the initial screen while divergent strains were only compared to the N2 wild-type group. Boxplots were generated using the ggplot2 (version 3.3.5) package.

*Classroom pedagogy*

We conducted the experiments described here across two laboratory sessions. These sessions were preceded with two training sessions where students learned how to conduct chemotaxis assays using known attractants (isoamyl alcohol) and repellants (carvone) (Ellington et al., 2020). An additional laboratory session involved conducting “field work” by having students construct aspirators and collect the ants needed for the divergent strain experiments. Weekly homework included reading relevant literature, data analysis and visualization, and writing the results and interpretations. The final project was to write this journal-style article.

## Reagents

**Table:** 

Strain Name	Genotype	Source
PD1074	Wild type	Caenorhabditis Genetics Center (CGC) at the University of Minnesota
CX10	*osm-9* ( *ky10* ) IV	Caenorhabditis Genetics Center (CGC) at the University of Minnesota
PR678	*tax-4* ( *p678* ) III	Caenorhabditis Genetics Center (CGC) at the University of Minnesota
N2 , BRC20067 , CX11271 , CX11276 , DL200 , DL226 , ECA246 , ECA36 , ED3040 , ED3048 , ED3049 , ED3052 , JU1088 , JU1172 , JU1213 , JU1242 , JU1440 , JU1568 , JU1581 , JU2007 , JU2466 , JU2519 , JU346 , JU360 , JU830 , KR314 , NIC1 , NIC252 , NIC256 , NIC277	Wild isolates; N2 (Bristol)	CeNDR at Northwestern University
